# Radcliffe Infirmary, Oxford

**Published:** 1894-12-15

**Authors:** John G. Ogle


### EDITOR'S LETTER-BOX.

### RADCLIFFE INFIRMARY, OXFORD.

In view of the letter published in The Hospital of December 1st, from the then resident medical officers at the Radcliffe Infirmary, we have communicated with those residents who have held office during the last few years, and have much pleasure in publishing the following letter from Dr. Ogle, dated from Reigate, December 4th, 1894:—

Sir,—In answer to your inquiry, I beg to say that I was house physician at the Radcliffe Infirmary, Oxford, from 1890 to 1892. During that time my relations with the House Committee as a whole were good, and, with many individual members of it, even cordial. My position and authority were assured, and on several occasions I was supported both by the medical staff and by the committee in certain needed reforms. No doubt under the system of management in force at the Radcliffe Infirmary there are opportunities for divided authority and the clashing of interests, which may not be so pronounced under other systems; but during my period of office the officials concerned rarely availed themselves of such opportunities. In fairness to the institution I trust you will find space to publish this in your next issue.—I am, faithfully yours,

John G. Ogle, M.D., Oxon.

